# ‘Seeing’ Strain in Soft Materials

**DOI:** 10.3390/molecules24030542

**Published:** 2019-02-01

**Authors:** Zhiyong Xia, Vanessa D. Alphonse, Doug B. Trigg, Tim P. Harrigan, Jeff M. Paulson, Quang T. Luong, Evan P. Lloyd, Meredith H. Barbee, Stephen L. Craig

**Affiliations:** 1 Applied Physics Laboratory, The Johns Hopkins University, Laurel, MD 20723, USA; vanessa.alphonse@jhuapl.edu (V.D.A.); Doug.trigg@jhuapl.edu (D.B.T.); Tim.harrigan@jhuapl.edu (T.P.H.); jeff.paulson@jhuapl.edu (J.M.P.); quang.luong@jhuapl.edu (Q.T.L.); Evan.lloyd@jhuapl.edu (E.P.L.); 2 Department of Chemistry, Duke University, Durham, NC 27708, USA; Meredith.barbee@duke.edu (M.H.B.); Stephen.craig@jhuapl.edu (S.L.C.)

**Keywords:** spiropyran, impact strain, poly(dimethyl siloxane), mechanophore, strain sensing

## Abstract

Several technologies can be used for measuring strains of soft materials under high rate impact conditions. These technologies include high speed tensile test, split Hopkinson pressure bar test, digital image correlation and high speed X-ray imaging. However, none of these existing technologies can produce a continuous 3D spatial strain distribution in the test specimen. Here we report a novel passive strain sensor based on poly(dimethyl siloxane) (PDMS) elastomer with covalently incorporated spiropyran (SP) mechanophore to measure impact induced strains. We have shown that the incorporation of SP into PDMS at 0.25 wt% level can adequately measure impact strains via color change under a high strain rate of 1500 s^−1^ within a fraction of a millisecond. Further, the color change is fully reversible and thus can be used repeatedly. This technology has a high potential to be used for quantifying brain strain for traumatic brain injury applications.

## 1. Introduction

Strains of soft materials at high rates can be characterized using several technologies including strain gauges and two dimensional digital image correlation (2D-DIC) during high-speed tensile test [1], split Hopkinson pressure bar test [2] and high-speed X-ray with lead tracers pre-embedded into the specimen [3]. These existing technologies suffer from a number of limitations when used for high rate testing. For example, the attachment of the strain gauges onto the specimen surface often alters the local stress field of the soft material resulting in inaccurate strain measurement [1]. Split Hopkinson pressure bar test requires the establishment of a uniform stress/strain field through the entire length of the specimen [2]. However, due to the low wave speed in soft materials, this is not always feasible resulting in poor data quality. 2D-DIC uses high speed cameras for capturing the surface features during the high rate process for strain calculation [4] but the interpolation function used for computing the strains can have a large effect on the bias errors of the matching leading to inaccurate measurement [5]. When 2D-DIC is used for soft materials, marking inks are often needed due to the lack of surface features and the inks need to be compatible with the specimen surface a condition that is not always possible. Lastly, high-speed X-ray requires very complex instrumentation and complicated pre-test sample tracer mounting for bulk strain measurement [3]. Most importantly, none of these existing technologies offers any direct measurements of the three dimensional (3D) spatial strain distribution inside the specimen during the impact event.

To directly visualize the 3D spatial strain distribution, we turned to the growing field of polymer mechanochemistry, specifically spiropyran (SP) mechanophore [6,7]. Upon mechanical stimulation, the sp^3^ spiro carbon-oxygen (C-O) bond in SP breaks leading to the formation of a longer, colored merocyanine (MC) [8,9,10]. By introducing ‘pulling points’ on both the chromene and indoline sides of the S-C-O junction, SP can be covalently bound to the matrix polymer, effectively coupling mechanical stress in the matrix polymer to the C-O bond. A number of polymer networks and structures have been demonstrated to exhibit mechanochromism [9,10,11,12], here we chose to focus on poly(dimethyl siloxane) (PDMS) for its tunability in mechanical properties, optical clarity and its wide use as impact simulants [13]. Alkene groups incorporated on both sides of the spiro junction allow easy covalent incorporation into commercially available PDMS kits, which form networks using platinum catalyzed hydrosilylation [11,12,13,14,15]. Since the transition between SP and MC is reversible, the color change in these polymer systems is fully recoverable and the SP-polymer matrix can thus be used multiple times for impact sensing.

Here we demonstrate the application of the SP mechanophores for sensing the impact strains in a PDMS elastomer, referred to as SP-PDMS hereafter. The objective of this work is to use SP mechanophore as a cost effective, passive strain sensor for measuring 3D spatial strains under impact conditions. Our ultimate goal is to use SP-PDMS as a brain surrogate for quantifying brain strain for traumatic brain injury (TBI), an injury that is responsible for 30% of all injury death in the Unites States alone [16]. The accurate impact strain measurement in brain surrogates can greatly help the design of impact helmet and further reduce TBI.

## 2. Experimental

### 2.1. Quasi-Static Tensile Test and Color Analysis

Quasi-static tensile testing was performed according to ASTM D412 with a die C geometry using a screw-driven MTS 30G at an extension rate of 0.36 mm/s at room temperature and the strain was measured via a strain gauge. During the tensile testing, a charge coupled device camera (Logitech Brio, Newark, CA, USA) was used to observe color change. The color of the quasi-static samples after stretching was deconvoluted into R, G, B values according to the method developed earlier [14]. Since the color of the specimen after deformation is primarily blue, we adopted a ‘blueness’ parameter for quantifying the color of each sample. The ‘blueness’ (B%) was computed by averaging the B value over the entire R, G, B scale, that is, B% = B/(R + G +B).

### 2.2. SP Synthesis

3′,3′-dimethyl-6-nitro-1′-(2-(pent-4-enoyloxy)ethyl)spiro[chromene-2,2′-indolin]-8-yl pent-4-enoate or spiropyran was synthesized according to [15] with the following modifications made to the amounts of reagents and purification methods. To an oven dried round bottom flask, 1′-(2-hydroxyethyl)-3′,3′-dimethyl-6-nitrospiro[chromene-2,2′-indolin]-8-ol (3.0 g, 8.14 mmol, 1 equiv) and 4-dimethylaminopyridine (0.099 g, 0.814 mmol, 0.1 equiv.) were dissolved in dry dichloromethane (40 mL). The dark green suspension was stirred and 4-pentenoic anhydride (3.20 mL, 17.51 mmol, 2.15 equiv) was added in 3 separate aliquots, with 15 min between each addition. The reaction was stirred overnight, resulting in a magenta-purple solution. The mixture was extracted with concentrated sodium bicarbonate solution (1 × 75 mL), 1 N hydrochloric acid (1 × 75 mL), water (2 × 75 mL) and brine (1 × 75 mL) before drying over sodium sulfate. The crude product was collected from rotary evaporation as crude purple oil. Boiling petroleum ether (300 mL) was poured into the oil, then the solution was hot filtered and let stand to develop yellow-green crystalline SP (3.21 g, 74%). Characterization matched the compound reported in literature [15].

### 2.3. SP-PDMS Elastomer Block Synthesis

An elastomer block was made with Sylgard^®^184 from Dow Corning and 0.25 wt% SP mechanophore [14]. SP was first dissolved in para-xylene at a concentration of 75 mg/mL followed by incorporating in the Sylgard^®^184 mixtures with different ratios of base to curing agent by volume (10:1, 20:1 and 30:1). The mixture was then degassed under vacuum for about 30 min until all gas bubbles were removed. The corresponding tensile and impact samples were then made by curing the mixers at ambient temperature for 48 h to ensure adequate formation of the network structure.

### 2.4. High Rate Impact Test and Finite Element Analysis

The high rate impact was performed using an in-house air cannon test system for achieving impact loading condition [17]. During the test, a domed cylindrical projectile (outer diameter 40 mm, length 52.5 mm, dome radius of curvature 20 mm and mass 29 g) made of 3D printed glass filled nylon was inserted into the air cannon barrel. The air pressure was then regulated to achieve a projectile impact speed from 80–110 m/s. Finite element analysis (FEA) used for predicting stress/strain distributions was performed using LS-Dyna based on parameters obtained from Mooney-Rivlin analysis of the quasi-static tensile test data. A Lagrangian formulation and an Euler forward time stepping algorithm were employed, with the time step size automatically chosen for numerical stability.

## 3. Results

### 3.1. Quasi-Static Test Results

**Figure 1:**
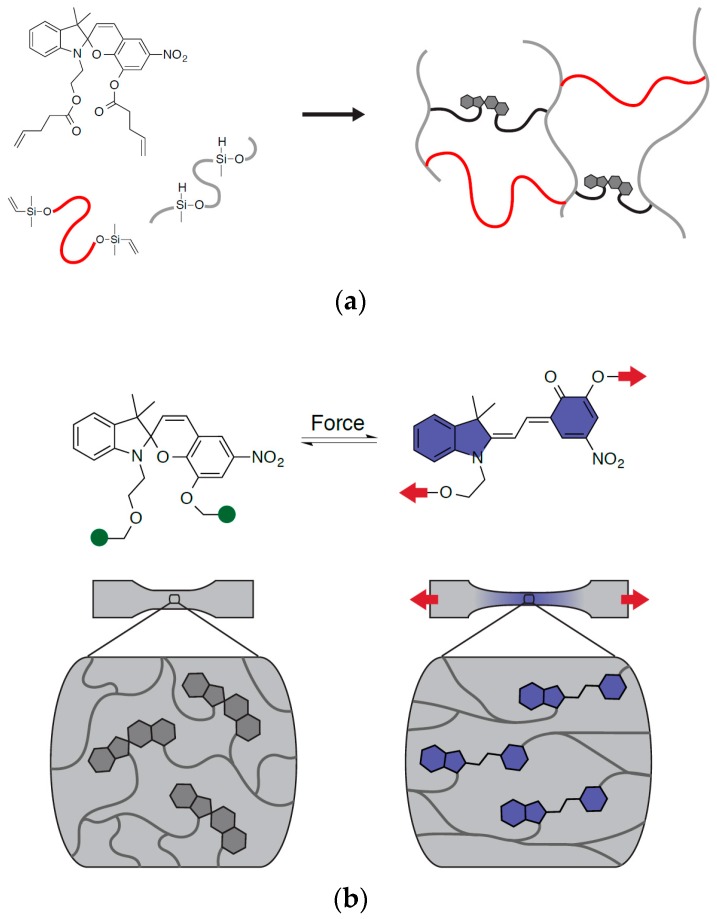
(**a**) Incorporation of SP (spiropyran) into PDMS (poly(dimethyl siloxane)) network structure. (**b**) Changing between SP and MC (merocyanine) during uniaxial mechanical deformation.

As discussed, the color change of SP relies on the rupture of the spiro C-O bond and the transformation of SP into the longer conjugated MC. The latter absorbs strongly at a wavelength of 550–600 nm and leads to the formation of purple color in SP-PDMS. Since MC is metastable, once the mechanical stimulus is removed, the spiro C-O bond will be reformed reversing MC into the original SP. The latter leads to the disappearance of the purple color and thus allows for multiple use. In this work, alkene functionalized SP [11,12,13,14,15] was used to couple with a vinyl terminated PDMS-Sylgard^®^ 184. The hydrosilylation reaction between Si-H and alkene groups lead to the formation of covalent bonding between SP and PDMS, resulting in the transfer of the load and thus color change upon impact (Figure 1).

**Figure 2:**
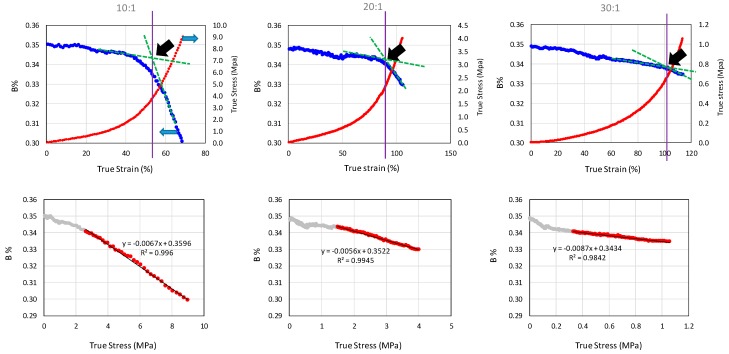
Effects of PDMS (poly(dimethyl siloxane)) mixing ratios on color change quasi-static tensile test. The top row shows blue color (B%) and true stress plotted against true strain and the dark arrows show the onset point for blue color change. The bottom row shows the B% plotted against true stress.

To understand the effect of PDMS network structure on the color change of SP, two part Sylgard^®^ 184 prepared at three different mixing ratios (10:1, 20:1 and 30:1) were tested under uniaxial tensile mode. Figure 2 shows the results at a strain rate of 0.014 s^−1^ for all three mixing ratios. In all three samples, the SP loading level was controlled at 0.25 wt%. It can be seen that the decrease in blue color (B%) initiated around the onset of the strain-hardening region. The onset point was determined by drawing lines tangent to the initial and final portions of the same curve with the interception of the two lines defined as the onset point. For the 10:1 mixing ratio, the onset true strain for the color change was found to be around 53%. With the increase in mixing ratio to 20:1 and 30:1, the onset true strain values for the color change increased to 92% and 102%, respectively. The bottom row of Figure 2 shows B% values plotted against true stress which demonstrates a close to linear relationship after the onset of strain hardening.

To better characterize PDMS network structure, the Mooney-Rivlin analysis was employed (Equation (1)) [18,19].

(1) $$\frac{\sigma_{\;engr}}{\left(\lambda -\frac{1}{\lambda^{2}}\right)}=2C_{1}+2C_{2}\frac{1}{\lambda }$$

where σ*_engr_* is engineering stress, *λ* is extension ratio, *C*_1_ and *C*_2_ are materials constants and relate to the network crosslinking density and the deviation from ideal rubber, respectively. *C*_1_ and *C*_2_ can be determined by plotting $\frac{\sigma_{\;engr}}{\left(\lambda -\frac{1}{\lambda^{2}}\right)}$ versus $\frac{1}{\lambda }$ with the slope and intercept being 2*C*_2_ and 2*C*_1_, respectively. The ratio of 2*C*_2_/*C*_1_ measures the looseness of the network structure [20]. With *C*_1_ and *C*_2_ known, the average molecular weight between crosslinks ($\bar{M_{c}}$) (kg/mol) and the network crosslinking density (*N*) (mol/m^3^) can be further determined via Equation (2).

(2) $$\bar{M_{c}}=\frac{\rho RT}{2C_{1}+2C_{2}}=\frac{\rho R}{\kappa N}$$

In the above equations, *κ* is the Boltzmann constant of 1.38 × 10^−23^ J/K, ρ is density (g/cm^3^), *T* is temperature (K) and *R* is the gas constant of 8.31 J/mol·K.

**Figure 3:**
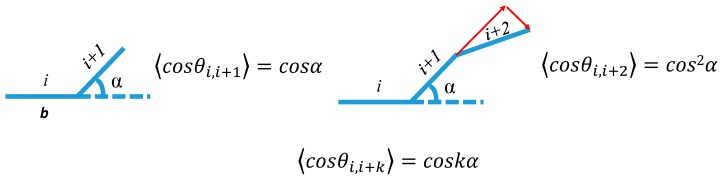
Schematic showing the calculation of end-to-end distance with fixed valence angles. The α is the bond angle and b is the bond length.

To better understand the SP-PDMS gelation network stretchability, the mean end-to-end distance (<*r*>) of PDMS chains using a fixed valence angle model (Figure 3) [20] was computed as follows.

$$\langle r^{2}\rangle =nb^{2}+2b^{2}\sum_{i=1}^{n}\sum_{k=1}^{n-i}\langle cos\theta_{i,\;i+k}\rangle =nb^{2}+2b^{2}\sum_{i=1}^{n}\sum_{k=1}^{n-i}cos^{k}\alpha \approx nb^{2}+2nb^{2}\frac{cos\alpha }{1-cos\alpha }=nb^{2}\frac{1-cos\alpha }{1+cos\alpha }$$

(3) $$\langle r^{2}\rangle =nb^{2}\frac{1-cos\alpha }{1+cos\alpha }$$

The force (*f*) that is required to perturb the chain dimensions can be shown as:

(4) $$f=\frac{3\kappa T}{Nb^{2}}r$$

where *n* is the number of repeat units between crosslinks, *b* is the Si-O bond distance of 1.63 Å and *α* is the bond angle of 50° [21].

Table 1 shows the computed results based on the Mooney-Rivlin analysis at a strain rate of 0.014 s^−1^. It can be seen that with the increase in mixing ratio from 10:1 to 30:1, $\bar{M_{c}}$ increased by a factor of twelve and *N* dropped by a factor of six indicating the formation of a looser network structures. Table 1 also shows the mean end-to-end distance (<*r*>) of PDMS computed using a fixed valence angle model (Equation (3)), which shows that with the increase of mixing ratio from 10:1 to 30:1, <*r*> increased by a factor of three.

To address the effect of network structure on the color change in SP, the theoretical maximum network stretch ratio (λ_t_) was calculated based on ratio between average chain contour length (τ) between crosslinks and <*r*>. These results are summarized in Table 2, which shows that with the increase in mixing ratio, the measured extension ratio (λ_m_) to λ_t_ decreased. Table 2 also shows the force (*f*) required to perturb the chains computed based on equation 4 decreased with the increase in <*r*> value. Since the color change in SP depends on the PDMS stretching induced spirocyclic C-O bond rupture, a smaller λ_m_/λ_t_ will lead to a lower stress (see last column in Table 2) being acted on the C-O bond causing less color change at a given strain. This finding is also consistent with the force calculation.

**Figure 4:**
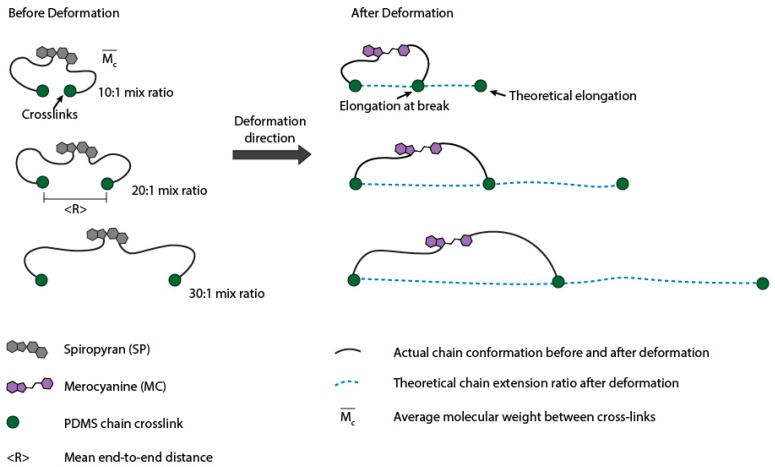
Schematic showing the chain contour length and mean end-to-end distance. Lower mixing ratio led to higher crosslinking density and thus smaller average molecular weight between crosslinks ($\bar{M_{c}}$).

A schematic on the effect of crosslinking density on the stretchability of the PDMS network is illustrated in Figure 4. Since high $\bar{M_{c}}$ means long ‘PDMS loop length’ between crosslinks, a high theoretical network stretchability (λ_t_) will ensue. At the same loading condition, the latter will compromise the effective stretching of SP and reduce the probability of transformation of SP into MC. As a result, at the same stretch ratio, the amount of the stress generated in PDMS with a higher mixing ratio will be much lower than that of a ‘tighter’ network structure. The latter in turn leads to higher extension for the onset of color change (λ_o_) (Table 2).

### 3.2. High Strain Rate Impact Results

**Figure 5:**
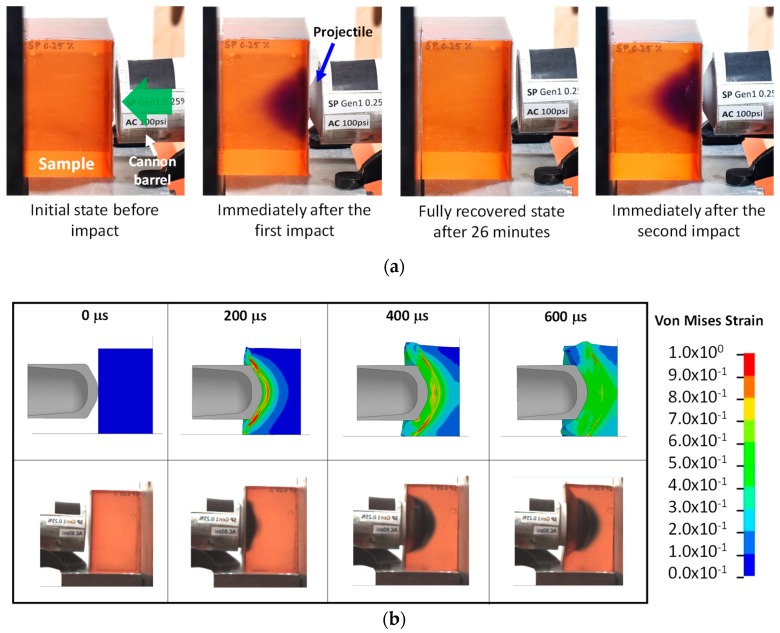
(**a**) Color change in SP-PDMS during and after impact at an impact speed of 100 m/s. The arrow shows the projectile impact direction. (**b**) Von Mises strain contours during the impact process via (top row) and high-speed video (bottom row). The PDMS (poly(dimethyl siloxane)) had a mixing ratio of 10:1 with 0.25% SP (spiropyran).

To further understand the high strain rate behavior, a SP-PDMS block (7.62 cm × 7.62 cm × 7.62 cm in size) with 10:1 mixing ratio was studied at impact speed ranging from 80–110 m/s. Figure 5a shows the representative color change in SP-PDMS before and after multiple impacts. Immediately after the first impact, a ‘purple cloud’ inside the block was formed showing the high strain contour during the impact. After about 26 min, the purple color fully disappeared indicating the switching back from MC to SP. A subsequent second impact had reformed the ‘purple cloud’ demonstrating the reusability of SP-PDMS for sensing impact strains.

**Figure 6:**
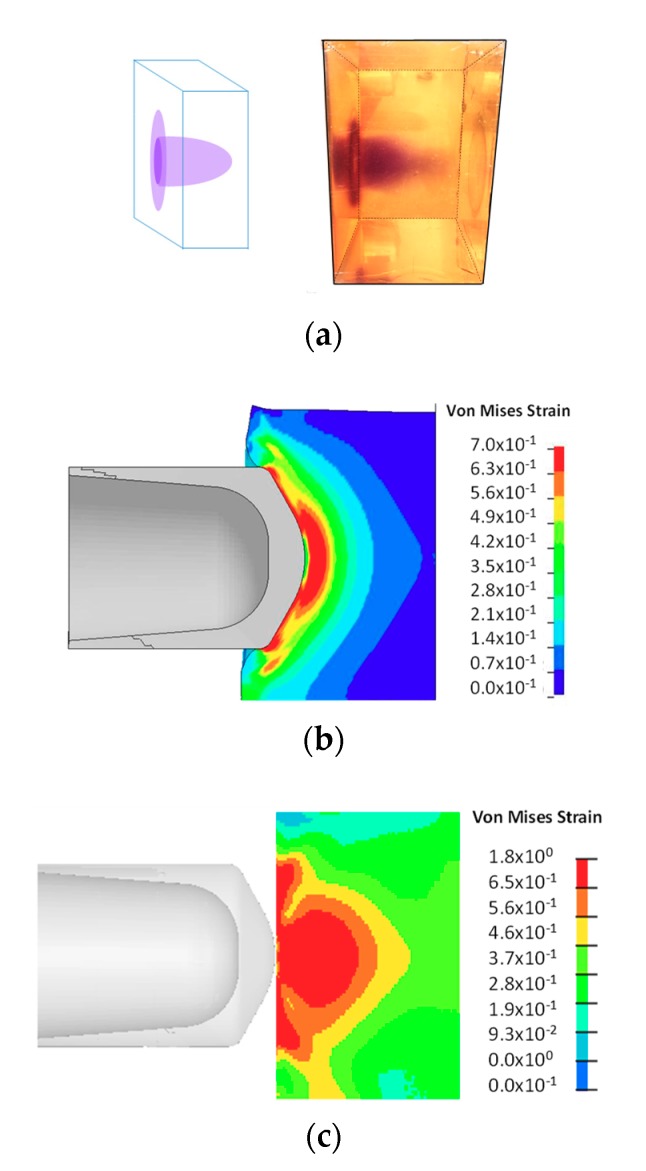
(**a**) Color change in SP-PDMS (spiropyran-poly(dimethyl siloxane)). (**b**) FEA shows the formation of the high strain areas on the surface of the specimen during the early impact. (**c**) Effective strain envelope based on FEA (finite element analysis) versus time. The strain levels are the maximum Von Mises strain that each material point experiences over the history of the impact. The highest strain locus is shown by the red color.

Figure 5b shows the progression of a projectile during impact. The formation of the high strain areas was evident. With the progression of the projectile, the maximum shear strain zone was pushed further into SP-PDMS block (Figure 5b top row). Figure 5b (bottom row) was taken during the impact process shows that formation of dark blue color during the impact. Note this color is different than the purple color observed in Figure 5a which was taken after the impact process. A similar finding on coloration was also found in Reference [15]. A detailed analysis of the high-speed impact video obtained at a frame rate of 30,000 per second showed that color change in SP-PDMS as fast as 33 µs was readily achieved during the ballistic impact event. Since the time frame of most impact event is in the neighborhood of microseconds, a 33 µs response time in SP-PDMS is thus fast enough for the SP to capture the impact induced damage in PDMS. We would also like to point out that the time response in SP is also affected by both the PDMS matrix viscoelastic properties and the impact rate as the load being transferred to the spiro junction is a direct function of the combined effects of both. A closer examination of SP-PDMS block after impact showed the presence of a purple surface ring (Figure 6a). This is because the low shear modulus of the SP-PDMS caused a very slow shear wave speed of 26 m/s ($\sqrt{\frac{G}{\rho }},$ where *G* is the shear modulus and *ρ* is the density). This shear wave speed was much slower than the bulk wave speed of 1461 m/s and prevented the relaxation of PDMS on the specimen surface during impact and led to the formation of high strain rings on the surface of SP-PDMS. This result was also confirmed by FEA (Figure 6b and Supplementary Material). Figure 6c shows the peak strain locus based on FEA envelope analysis throughout the whole impact process.

### 3.3. Application

Here, we have demonstrated that SP-PDMS elastomer block can be used for ‘seeing’ the dynamic impact strain under ballistic conditions. By examining the color distribution in the SP-PDMS, an overall 3D spatial strain contour can be established. Since the color change can ‘remember’ the highest strain regions (Figure 6c) for the same impact duration, this technology can be used to visualize the highest strain levels in a test sample. This latter finding, if used for traumatic brain injury (TBI) application, can be used for sensing the regions susceptible to brain injury whereas none of the existing technologies can do. In TBI, it has been shown that the most effective strategy in reducing brain injury is the use of impact resistant helmets [22,23]. As a result, one way of adopting this technology is to use SP-PDMS as a brain surrogate for TBI protecting helmet evaluation. By comparing the SP-PDMS color during impact to the relationship established between color and strain under quasi-static conditions (Figure 2), a continuous strain distribution in the PDMS can then be established. The latter further enables the construction of aa spatial impact strain distribution in the brain surrogate. Since this technology does not require the use of any pressure sensors which only measure impact at discrete locations, the overall strain spatial distribution inside the brain surrogate can be obtained, allowing the visualization of impact induced brain strain in situ. A color holding time of 26 min is also desirable and can allow for post impact brain surrogate imaging and damage analysis. Finally, we would like to point out that although this work opens a potential new way of visualization of brain strain during high rate impact, the sensitivity of SP is low and needs to be improved. For example, the typical strains for TBI is about 5–7% [4], whereas the lowest onset strain for the color change in three SP-PDMS blocks with three different mixing ratios was 53% and this value is 10 times of the TBI strain. In addition, the moduli of the PDMS blocks is also much higher than that of a human brain [24,25]. As a result, future efforts will be directed toward the use of softer elastomers that have better biofidelity to human brains. The path to such materials will likely involve two complementary approaches: (i) the design and synthesis of polymer networks that are both soft and still direct macroscopic strain to mechanochromic molecules such as spiropyran; and, (ii) the design and synthesis of mechanophores that give measurable response at even lower strains. One strategy toward the latter objective may include the introduction of more electron withdrawing groups on the chromene side of SP or the addition of “lever arm” effects that create greater mechanical advantage at the molecular level [26,27].

## 4. Conclusions

We have shown that the combination of alkene modified SP mechanophore with a vinyl terminated PDMS elastomer can be used for visualizing the impact strain in a PDMS elastomer via monitoring color change of SP in PDMS. Since the color change is an indication of the highest strain levels in the brain simulant, this technology can thus provide a 3D continuous spatial distribution of strain level in a test specimen, whereas conventional technologies can only provide discrete measurements. Further, the reversible color change in SP allows the repeated use of SP-PDMS for impact evaluation. Lastly, this technology also showed promising potential for sensing brain injury in a brain surrogate material.

## Figures and Tables

**Table 1:** Effect of mixing ratio on the structure and performance of PDMS network structure. Tensile test strain rate was 0.014 s^−1^.

Sylgard^®^ 184 Mixing Ratio	C1 (kPa)	C2 (kPa)	$\bar{M_{c}}\;(\mathrm{kg}/\mathrm{mol})$	N (mol/m^3^)	Onset True Strain for Blue Color (B%) Change	Root Mean End-to-End Distance (<*r*>) (Å)
10:1	611	189	1.6	646	53%	9.7
20:1	168	123	4.4	235	92%	16.1
30:1	99	30	19.5	104	102%	33.9

**Table 2:** The effect of network structure on color change and network stretchability.

Slygard^®^ 184 Mix Ratio	Chain Contour Length between Crosslinks (τ) (Å)	Maximum Theoretical Extension Ratio (λ_t_ = τ/<R>)	Measured Extension Ratio (λ_m_)	Observed Elongation for the Onset of Blue Color Change (λ_o_)	Percent Reached to Theoretical Extension Ratio (λ_m_/λ_t_)	Force Required to Perturb the Chain Dimensions (f) (picoNewton-pN)
10:1	35.1	3.6	2.0	1.6	56%	20.9
20:1	96.4	6.0	2.9	2.4	49%	12.6
30:1	429.4	12.7	3.1	2.7	25%	6.0

## Supplementary Materials

The following are available online, Video S1: Comparison between high speed impact (left) and finite element analysis (right).
